# *Pseudomonas* in Meat Processing Environments

**DOI:** 10.3390/foods14091615

**Published:** 2025-05-02

**Authors:** Chloe Calhoun, Ifigenia Geornaras, Peipei Zhang

**Affiliations:** Department of Animal Sciences, Colorado State University, Fort Collins, CO 80523, USA; chloe.calhoun@colostate.edu (C.C.); gina.geornaras@colostate.edu (I.G.)

**Keywords:** *Pseudomonas*, meat production, biofilms, Shiga toxin-producing *E. coli*, *Salmonella*, *Listeria monocytogenes*

## Abstract

*Pseudomonas* is often predominant and/or prevalent among the residential microbiota in food processing facilities and has the potential to enhance the survival of pathogenic bacteria in these environments. This review aims to discuss our current understanding of this bacterial genus in meat processing environments. We specifically focus on the predominant species of *Pseudomonas* in meat and meat processing plants, their biofilm-forming abilities and affecting factors, and the interaction between *Pseudomonas* and foodborne pathogens in biofilms. Published accounts indicate *Pseudomonas* has more diversity within and between meat plants compared to that in meat products. Despite the competition between *Pseudomonas* and other bacteria, including pathogens, this genus can increase the survival of pathogenic bacteria in food processing-related environments by increasing the resistance of pathogens to antimicrobials when present in biofilms. Our understanding of the ecology of *Pseudomonas* in meat processing environments needs further exploration. Future studies should consider biofilms formed under dynamic conditions simulating meat plant operations, using species/strains that are more representative of the *Pseudomonas* populations found in meat processing environments, and with growth media that closely resemble the nutrients found on meat processing surfaces.

## 1. Introduction

Tremendous efforts have been put into cleaning and sanitizing the production environment in meat processing facilities. However, bacteria, including foodborne pathogens, are still able to survive and persist in the relevant environments leading to potential contamination of meat products and consequently food safety issues [1,2,3,4]. Meat products are often implicated in human infections caused by foodborne pathogens such as *Salmonella*, Shiga toxin-producing *Escherichia coli* (STEC), *Listeria monocytogenes*, and *Campylobacter* [5]. Biofilms formed by meat plant bacteria in meat processing facilities can play an important role in the persistence of foodborne pathogens. Pathogenic bacteria, regardless of their biofilm-forming abilities, are able to be incorporated into the biofilms developed by meat plant bacteria [6,7], which likely contribute to their survival in the harsh meat processing environments. Among the microbiota present in meat plants, *Pseudomonas* has often been reported to be the most prevalent and/or predominant genus [8,9,10]. *Pseudomonas* spp. can affect meat quality due to their well-known spoilage activities. Certain *Pseudomonas* species can produce lipases and proteases, leading to the release of free fatty acids and amino acids and further resulting in off-odors and rancidity of meat products [11]. Their spoilage activities are also ascribable to the abilities of producing extracellular slime and pigmentation growth in meat [11]. This bacterial genus may also affect meat safety considering its predominant/prevalent status among residential meat plant microbiota and its potential to enhance the survival of foodborne pathogens in biofilms.

In this mini review, we aim to discuss the current progress in our understanding of *Pseudomonas*, a bacterial genus that can affect meat quality and potentially indirectly affect meat safety. With a focus on plants processing raw red meat products, we specifically cover the topics including *Pseudomonas* species in meat products and meat processing environments, the biofilm-forming abilities of *Pseudomonas*, the factors affecting biofilm formation by *Pseudomonas*, and the potential effect of *Pseudomonas* biofilms on the survival of foodborne pathogens.

For each topic included in this review, we searched for the relevant published literature in Web of Science. The phrases used for searching included “*Pseudomonas*”, “*Pseudomonas* in meat products”, “*Pseudomonas* in meat plants”, “*Pseudomonas* in meat production environments”, “*Pseudomonas* in meat processing plants”, and “*Pseudomonas*, biofilm”. Due to the limitation of the search engine in determining relevance, >500 publications often showed up in the search results for a phrase we used. Therefore, we either reviewed the first 500 most relevant articles or all the articles if the total number was <500, to manually select those related to our topics.

## 2. The Habitat of *Pseudomonas*

*Pseudomonas* belongs to the family of *Pseudomonadaceae* and is Gram-negative, obligately aerobic, and mostly motile [12]. There are a large number of species in this genus and the number of newly identified species is still steadily increasing. As of 22 January 2025, 350 species with a validly published and correct name have been reported (https://lpsn.dsmz.de/genus/pseudomonas). Depending on the species/strains, *Pseudomonas* can grow at temperatures ranging from 4 to 45 °C [12], and can live and thrive in a wide range of aquatic and terrestrial habitats [12,13]. *Pseudomonas* spp. have been found in water [14,15], soil [15,16,17,18,19], and sediment [20,21,22,23], and have also been recovered from plants [24,25,26,27,28,29] and animals [30,31,32,33]. Some *Pseudomonas* can be plant growth promotors [34,35,36,37], while others are pathogenic to plants [38,39,40]. Humans can also serve as hosts for *Pseudomonas*, with *P. aeruginosa* being a notable nosocomial pathogen [41]. Additionally, strains of other species, such as *P. putida* [42], *P. fluorescens* [43], *P. monteilii* [44,45,46], *P. fulva* [47], *P. nosocomialis* [48], *P. pharyngitis* [49], *P. asiatica* [46], and *P. plecoglossicida* [50] have occasionally been reported as human pathogens or have been isolated from patients in hospital [51].

Moreover, *Pseudomonas* spp. are well-known spoilage microorganisms in many foods [11], such as raw milk, fish, and meat products. The surfaces of food processing facilities can be an important source of bacteria contaminating meat products [1,2,52,53]. *Pseudomonas* has often been reported as prevalent and/or predominant in these environments [8,9,10].

### 2.1. Pseudomonas Found in Meat Products

Similar to many other perishable food types, *Pseudomonas* is frequently recovered from raw meat products and is believed to be one of the major players associated with meat spoilage, particularly in oxygen-packaged products [54,55]. Numerous studies have reported the overall predominant status of *Pseudomonas* in meat as a genus, while there are fewer studies that have identified *Pseudomonas* at the species level [15,52,54,55,56,57,58,59,60,61,62,63,64,65]. Species-level characterizations have mainly used methods with a limited resolution level for *Pseudomonas* species differentiation. Such methods include biochemical tests [15,54,56,57,58], 16S rRNA gene sequencing [52,59,60,61,62,63], RNA-based amplicon sequencing [65], denaturing gradient gel electrophoresis (DGGE) [61,64], restriction fragment length polymorphism analysis (RFLP) [59], matrix-assisted laser desorption/ionization time-of-flight mass spectrometry (MALDI-TOF MS) [55], and PCR [61] (Table S1). Most of these studies identified *P. fragi* as the predominant *Pseudomonas* species in meat products in air packaging (AP) or modified atmosphere packaging (MAP) containing ≥50% O_2_, regardless of whether the meat was fresh or spoiled. *P. fluorescens* has been the second most often detected *Pseudomonas* species among these studies, and *P. lundensis*, *P. putida*, *P. psychrophila*, and *P. weihenstephanensis* have been occasionally reported.

### 2.2. Pseudomonas in Meat Processing Plants

Microbiota in meat processing environments have been investigated using either culture-dependent methods or high-throughput sequencing approaches [6,9,52,66,67,68,69,70,71,72,73,74,75,76,77,78,79,80,81,82,83,84,85,86,87,88,89,90,91] (Table S2). Most of these studies, if not all, have shown that *Pseudomonas* is either the most predominant genus or one of the most predominant genera among meat plant bacterial communities. *P. fragi* and *P. fluorescens*, the top two most often detected *Pseudomonas* species in meat products, are frequently found in meat processing plants as well (Table S2), indicating that environments associated with meat production are potential sources of meat product contamination. In contrast to the studies on meat products, which have pointed to a limited number of species as the predominant ones among *Pseudomonas* (Table S1), the species of this bacterial genus found in meat plants showed relatively greater diversity and inconsistency across studies (Table S2). For example, Yang et al. [91] investigated the microbiota in a beef fabrication facility in the U.S. and the sequencing data showed *P. proteolytica*, *P. fragi*, *Pseudomonas* sp. TMW 2.1634, *P. aeruginosa*, and *P. psychrophila* were the top five most predominant species of *Pseudomonas* [10]. Nevertheless, in the study by Alvarez-Molina et al. [83], *P. fluorescens*, *P. fragi*, *P. putida*, *P. lundensis*, and *P. psychrophila* were the top five most predominant *Pseudomonas* species.

Factors such as the sanitation practices among meat plants investigated, the source of processed animal species, geographical regions of the plants, and the target functional areas in the plants may all influence which species/strains of *Pseudomonas* will survive and thrive in a meat processing facility, contributing to the inconsistencies across studies. There is a limited understanding of how these factors affect *Pseudomonas* populations in meat plants. However, such information may help us develop more effective measures to control this group of bacteria and improve both the safety and quality of the meat products produced.

### 2.3. Discrepancy in Species Identification of Pseudomonas

With the advancement of next- and third-generation sequencing technology, it has become a standard to use whole-genome-based methods, including average nucleotide identity (ANI) and digital DNA–DNA hybridization (dDDH), to identify bacteria, including the species among *Pseudomonas* [92]. Discrepancies in species identity between conventional methods and whole-genome-based methods have frequently been reported for *Pseudomonas*. For example, in the study by Lick et al. [93], 16S rRNA gene sequencing was not able to differentiate between a *Pseudomonas* strain V3/3/4/13^T^ and *P. fragi*; however, ANI and dDDH showed this strain belonged to a different species, for which a new species name, *P. kulmbachensis*, was proposed. Stanborough et al. [94] performed whole genome analysis on 12 *P. fragi* and seven *P. lundensis* isolates recovered from meat and milk, the identities of which were originally confirmed using *Pseudomonas* species-specific PCR. The analysis revealed that these isolates likely belong to more than two species because the ANI between certain isolate combinations within each ‘so-called’ species was <96%, the threshold recommended for species delineation [92]. Four new species were proposed by analyzing the genomes of *P. putida* strains, originally defined using 16S rRNA gene sequencing [18]. Specifically, these species included *P. alloputida*, *P. inefficax*, *P. persica*, and *P. shirazica* [18]. Recently, Sequino et al. [76] used metagenome sequencing to characterize the microbial composition in beef samples stored in air packaging and found that the predominant *Pseudomonas* species in these samples were *P. paraversuta*, *P. versuta*, and *P. fragi*. The former two species were proposed as new species only in the last few recent years, with *P. paraversuta* in 2021 and *P. versuta* in 2017 [95,96]. As such, whole-genome-based methods are recommended in future studies characterizing *Pseudomonas*, regardless of those in meat products or meat processing plants.

## 3. Biofilm-Forming Abilities of *Pseudomonas*

A biofilm is an organized community of microbial cells embedded in a highly hydrated matrix of extracellular polymeric substances (EPSs), which can form on biotic or abiotic surfaces [97]. Polysaccharides/carbohydrates, proteins, extracellular DNA (eDNA), and lipids form the main components of EPSs [98]. Bacteria in biofilms are primarily protected by EPSs which act as a physical barrier by diffusing or capturing antimicrobial molecules [99]. Biofilm formation on surfaces is believed to be one of the main strategies by which bacteria persist in food processing facilities [100]. Sanitizers at in-use concentrations in food processing facilities are frequently reported to be ineffective against bacteria in biofilms [101].

Studies investigating biofilm development by *Pseudomonas* originating from raw meat products or raw meat processing facilities are limited. However, studies on *Pseudomonas* from other food types or food processing plants may offer insights into the behavior of *Pseudomonas* in meat processing environments. Therefore, we extended the scope of our literature search to include available publications investigating *Pseudomonas* from products and processing environments of both raw red meat and many other food types such as chicken, seafood, milk, dairy products, and vegetables. In these studies [7,102,103,104,105,106,107,108,109,110,111,112,113,114,115,116,117,118,119,120,121,122,123,124,125,126,127], biofilms were grown on stainless steel or plastic surfaces (polystyrene or polyvinyl chloride coupons, or polystyrene microtiter plates) and were analyzed for viable bacterial numbers, quantified for total biomass using the crystal violet (CV) staining method, and/or measured for specific EPS component.

### 3.1. Pseudomonas Species Are Often Reported as Strong Biofilm Formers Compared to Other Bacterial Species

Various species of *Pseudomonas* have been reported as biofilm formers [102,103,104,105,106,107,108]. For example, *P. fluorescens*, *P. fragi*, and *P. putida* strains isolated from chicken meat or a chicken conveyor belt were able to develop biofilms on polystyrene surfaces at 20 °C and/or 30 °C depending on the strain [107]. Wang et al. [106] reported a bacterial cell density of 9.6 Log CFU/cm^2^ for the biofilm formed on a stainless steel surface at 20 °C by a *P. fluorescens* strain H5 isolated from a spoiled chicken carcass, and the thickness of the biofilm was up to 120 µm. Yushina et al. [102] investigated the biofilm-forming ability of 22 strains belonging to 22 *Pseudomonas* species, including *P. proteolytica*, *P. putida*, *P. fragi*, and *P. koreensis*, in microtiter plates incubated at 37 °C. All the strains included were classified as strong biofilm formers based on the CV staining method (OD_540_ > 1.0 after 72 h incubation) [102].

*Pseudomonas* has been frequently compared to other bacterial species, including both spoilage-associated microorganisms and pathogens, regarding its ability to form biofilms. Although sporadic strains were not able to develop biofilms stronger than others [107], *Pseudomonas* strains often had higher bacterial numbers and/or greater EPS production than many other bacterial species or genera in biofilms [112,115,118,122].

Wagner et al. [115] evaluated the biofilms developed on stainless steel in tryptic soy broth (TSB), incubated at 10 °C for 7 days, by 11 individual bacterial strains recovered from a meat processing plant. These strains included *P. fragi* BF1, *Acinetobacter harbinensis* BF1, *Arthrobacter* sp. BF1, *Brochothrix thermosphacta* BF1, *Carnobacterium maltaromaticum* BF1, *Kocuria salsicia* BF1, *Lactococcus piscium* BF1, *Microbacterium* sp. BF1, *Psychrobacter* sp. BF1, *Rhodococcus erythropolis* BF1, and *Stenotrophomonas* sp. BF1. *P. fragi* had the second largest bacterial load (7.3 Log CFU/cm^2^), with *Microbacterium* sp. BF1 having the largest (8.7 Log CFU/cm^2^). Furthermore, the *P. fragi* strain was one of six species in which all three components of EPSs, namely carbohydrates, eDNA, and protein, were detected [115]. In the study by Papaioannou et al. [118], biofilms were grown for 6 days at 15 °C on stainless steel coupons in periodically renewed model fish juice substrate simulating fish processing conditions. A cocktail of four strains belonging to four *Pseudomonas* species (*P. fragi*, *P. fluorescens*, *P. putida*, and *P. savastanoi*) had higher bacterial numbers in biofilms developed than those in the biofilms developed by a cocktail of six *L. monocytogenes* strains. Pang et al. [122] grew single-species biofilms with *P. aeruginosa*, *Salmonella* Typhimurium, and *Salmonella* Enteritidis on stainless steel coupons submerged in TSB at 25 °C for 6 days and found that the *P. aeruginosa* strain produced the highest amount of carbohydrates and proteins in its biofilms. A *P. fluorescens* strain PF07 was reported to have both higher bacterial cell numbers and higher amounts of extracellular polysaccharide than a *Shewanella baltica* strain in single-species biofilms grown in polystyrene microtiter plates incubated at 4, 15, and 30 °C [112].

Biofilms in meat production environments are typically composed of multiple bacterial species. *Pseudomonas* often predominates over other bacterial species, such as *E. coli* O157:H7, *Salmonella*, *L. monocytogenes*, and spoilage-associated microorganisms in dual- or multiple-species biofilms grown under laboratory conditions simulating the food production [103,116,117,118,119]. This aligns well with the predominant status of *Pseudomonas* in food processing facilities as previously reviewed in the published literature [8,9,10].

### 3.2. Factors Affecting Biofilm Formation by Pseudomonas

The biomass of biofilms increases following attachment of bacterial cells to a surface and the increase continues until the biofilm matures, after which point it may decrease if dispersion occurs [110,112,113]. The time taken for a biofilm to mature differs at different temperatures with a longer time generally required at lower temperatures [110,112,113]. However, chilled temperatures seem to be able to stimulate maximum biofilm production by certain species/strains of *Pseudomonas* on plastic surfaces. Studies investigated biofilm development of *Pseudomonas* on surfaces such as 96-well polystyrene plates (*P. lundensis*, [110]), polyvinyl chloride coupons (*P. putida*, [113]), and 24-well polystyrene plates (*P. fluorescens*, [112]), and used CV staining to quantify the biofilm biomass. These studies reported that the maximum biomass reached during the whole biofilm development process was larger at 4 and 10 °C than at 30 °C [110], at 5 and 10 °C than at 20 and 30 °C [113], and 4 and 15 °C than at 30 °C [112]. Zhu et al. [112] also reported much higher levels of exopolysaccharide in mature biofilms of *P. fluorescens* formed at 4 °C than at 15 °C. However, in these studies, viable bacterial cell numbers of the biofilms were not determined.

There are also studies comparing the amount of biofilm formed at different temperatures at certain times without considering the biofilm development stage. The results of these studies have been inconsistent. Zhu et al. [111] tested biofilm formation by *P. lundensis* PL28 in 96-well polystyrene microtiter plates and found that the total biomass quantified via CV staining was higher at 15 °C than at 30 °C regardless of incubation time (24 and 48 h). On the contrary, Sternisa et al. [109] reported that the biomass of biofilms was higher at warm (30 °C) than chilled temperatures (15 and 5 °C) for both 72 h biofilms developed by a *P. fragi* strain and a *P. psychrophila* strain individually in 96-well polystyrene microtiter plates. However, no difference was observed for 24 h biofilms in this study, which may be because biofilms by both strains were still growing at this time point and had not developed enough biomass to make the difference.

Also, different *Pseudomonas* strains may vary in their responses to temperature when forming biofilms. Four *Pseudomonas* isolates, including two *P. putida* (strains I and II), one *P. fragi*, and one *P. fluorescens*, were included in the study by Ning et al. [107]. *P. putida* I showed a numerically larger amount of biofilm biomass at 20 °C than at 30 °C regardless of incubation time (24 and 72 h). However, the other three *Pseudomonas* isolates had a numerically larger amount of biofilm at 20 °C than at 30 °C after 24 h of incubation, whereas the opposite trend was noted after 72 h. A vegetable-associated *Pseudomonas* sp. was used to grow biofilms on polystyrene coupons at 5 and 30 °C for 5 days [120]. The obtained biofilms had both higher bacterial counts and a larger thickness at 30 °C than at 5 °C.

The temperature dependency of biofilm forming abilities by *Pseudomonas* on stainless steel surfaces rather than plastic surfaces has also been investigated in a limited number of studies, but mainly without considering the development stage of the biofilm. The findings of these studies have also been inconsistent. At 72 h, the biomass in biofilms formed by *P. fragi* ZM648 and *P. psychrophila* ZM652 on stainless steel coupons decreased as the incubation temperature (30, 15, and 5 °C) decreased [109]. However, the biomass in 24 h biofilms did not show a difference at different temperatures. Also, bacterial numbers in biofilms did not show a temperature dependency for either incubation time. In another study [114], researchers examined *P. fragi* ATCC 4973 biofilm formation on stainless steel coupons at 5 and 15 °C and measured the biomass after 24, 72, and 168 h of incubation. The comparison of biomass between the two temperatures varied with incubation time, with a larger biomass at 15 °C than at 5 °C at 24 and 72 h, while the opposite was observed at 168 h.

Overall, the findings of the studies described above indicate that temperature and other factors, such as the materials on which biofilms are developed, and incubation time (i.e., stage of biofilm), can all influence the biomass of *Pseudomonas* biofilms. Additionally, it is important to note that the use of different strains in these studies can also be a factor for the variation in biofilm formation.

### 3.3. Pseudomonas Biofilms and Foodborne Pathogens

Biofilms developed by food plant bacteria are potentially able to provide shelter for pathogenic bacteria and contribute to their persistence in food processing facilities. Although *Pseudomonas* is often more competitive than other bacterial species, including foodborne pathogens, in biofilms as discussed above, it can potentially enhance the survival of pathogens in food processing environments.

A Shiga toxin-producing *E. coli* O157:H7 strain was not able to form biofilm on its own; however, it was able to insert itself into the biofilms developed by meat plant bacteria that were predominated by *Pseudomonas* [6]. The published literature has investigated the susceptibility of *Salmonella* to sanitizers in single-species and dual-species biofilms formed with *Pseudomonas* [121,122,123]. These studies found a significant difference in the resistance of *Salmonella* to sanitizers between single- and dual-species biofilms at a “young age”, although the difference often disappeared as biofilms “aged” and became more resistant. In the study by Leriche and Carpentier [123], when exposed to 5.5 mg/L free chlorine, reductions of a *Salmonella* Typhimurium strain in a 1-day-old single-species biofilm (3.3 Log CFU) were greater than when it was in a dual-species biofilm formed with a *P. fluorescence* strain (2.8 Log CFU). A similar trend was observed for 4-day biofilms, suggesting that the presence of *P. fluorescens* resulted in an increased tolerance of *S.* Typhimurium to chlorine [123]. Pang et al. [122] grew biofilms with a *Salmonella* Enteritidis strain either alone or in dual species with a *P. aeruginosa* strain. Following exposure to Whisper^TM^ V (a blend of four effective quaternary ammonium compounds) on day 2, *S.* Enteritidis in the dual-species biofilm was reduced by 3.0 Log CFU/cm^2^, which was less than the reduction obtained when in the single-species biofilm (3.7 Log CFU/cm^2^). When 2-day-old dual- and single-species biofilms were treated with chlorine (50 µg/mL), *S.* Enteritidis was reduced by 3.0 and 5.4 Log CFU/cm^2^, respectively. The authors also made microscopic observations and found EPSs covering large microcolonies in the dual-species biofilms but not in mono-species ones. The same research laboratory [121] further grew biofilms for the two bacterial strains in chicken juice and observed a similar protective effect by the *P. aeruginosa* strain.

Enhanced survival under harsh conditions due to *Pseudomonas* in mixed-species biofilms compared to single-species biofilms has also been reported for *L. monocytogenes*. Haddad et al. [124] grew biofilms on stainless steel or high-density-polyethylene (PE-HD) coupons using *L. monocytogenes* Lm568 alone or with *P. fluorescens*. To simulate meat processing conditions, the biofilms were developed under a 12-day regimen that included periods of nutrient cycling and starvation. *L. monocytogenes* showed an increased survival of 0.5–1.8 Log on SS or PE-HD coupons in the presence of *P. fluorescens* compared to mono-culture biofilms. Also, in biofilms with *P. fluorescens*, *L. monocytogenes* showed a significantly higher minimum biofilm eradication concentration (MBEC). The protective effect provided by *Pseudomonas* to *L. monocytogenes* against antimicrobial interventions has also been reported in a few other studies, including that of *P. lundensis* to ε-polylysine hydrochloride [111], *P. fluorescens* to chitosan [125], and *P. fragi* to PVDF-HFP/PVP/MoO3 nanocomposite [114]. On the contrary, Giaouris et al. [126] grew mono- and dual-species biofilms with three strains of *L. monocytogenes* and/or three strains of *P. putida* on stainless steel coupons at 18 °C for 10 days. They did not find a difference in the tolerance of *L. monocytogenes* to benzalkonium chloride between the biofilms with and without *P. putida* [126].

In addition, it has been reported by a limited number of studies that pre-colonization by *Pseudomonas* on a surface can enhance the attachment by other bacterial species. In a study conducted by Castonguay et al. [7], an *E. coli* K-12 strain was unable to attach to solid surfaces and grow a biofilm, but when it was mixed with *P. putida*, a mixed-species biofilm was formed. In the study by Hassan et al. [127], pre-colonization by *P. putida* enhanced the attachment of *L. monocytogenes*.

### 3.4. Overview

Altogether, considering the limited amount of research conducted on *Pseudomonas* species isolated from meat or meat production, we included in our review biofilm studies involving *Pseudomonas* from various other food types and processing environments. These studies have shown that *Pseudomonas* strains often predominate over many other bacterial species, including pathogens, in biofilms. Despite the competition between *Pseudomonas* and others, members of this genus can increase the survival of pathogenic bacteria in food processing-related environments by increasing the resistance of pathogens to antimicrobials and/or enhancing the attachment of pathogens to processing surfaces. Several factors can affect the biofilm-forming abilities of *Pseudomonas*, including the temperature, the materials on which biofilms are developed, and the strains included for study.

*Pseudomonas* may show similar behavior in environments processing meat and other food types. However, we should recognize that more studies related to *Pseudomonas* from meat products and meat production environments under meat processing conditions are warranted. In addition, the vast majority of studies included in our discussion used laboratory media such as TSB or food matrices other than meat to grow biofilms. This may not be able to represent the nutrients encountered by bacteria in meat processing facilities, the surfaces of which are likely contaminated with, or were once in contact with, meat debris or fat. As such, better medium options should be explored. Also, *Pseudomonas* biofilm studies have been mainly conducted under static conditions with constant temperature and once-added nutrients. A 24 h production cycle in meat processing facilities generally has fluctuations in temperature, with downtime periods typically experiencing higher temperatures than those during operation. Also, the environmental surfaces in meat processing plants likely have different nutrient levels before and after cleaning and sanitation. Furthermore, considering the large diversity of *Pseudomonas* between and within meat processing plants, more species/strains should be included in studies to improve our understanding of the ecology of *Pseudomonas* in meat processing environments.

Our review has mainly focused on *Pseudomonas*, a bacterial genus often reported as predominant/prevalent in meat processing environments. However, we recognize that many other species, such as *Psychrobacter* and *Acinetobacter*, are often found in meat production environments as well [10]. These bacteria may also play a role in biofilm development and affect the survival of pathogens in the relevant environments.

## Supplementary Materials

The following supporting information can be downloaded at: https://www.mdpi.com/article/10.3390/foods14091615/s1, Table S1: Published studies with species of *Pseudomonas* reported in raw red meat products including beef, pork, and lamb; Table S2: Predominant *Pseudomonas* species in plants processing raw red meat products including beef, pork, and lamb.
